# Thermal adaptation of decomposer communities in warming soils

**DOI:** 10.3389/fmicb.2013.00333

**Published:** 2013-11-12

**Authors:** Mark A. Bradford

**Affiliations:** School of Forestry and Environmental Studies, Yale UniversityNew Haven, CT, USA

**Keywords:** carbon use efficiency, climate warming, microbial growth, modeling, soil respiration, review, soil organic matter, thermal acclimation

## Abstract

Temperature regulates the rate of biogeochemical cycles. One way it does so is through control of microbial metabolism. Warming effects on metabolism change with time as physiology adjusts to the new temperature. I here propose that such thermal adaptation is observed in soil microbial respiration and growth, as the result of universal evolutionary trade-offs between the structure and function of both enzymes and membranes. I review the basis for these trade-offs and show that they, like substrate depletion, are plausible mechanisms explaining soil respiration responses to warming. I argue that controversies over whether soil microbes adapt to warming stem from disregarding the evolutionary physiology of cellular metabolism, and confusion arising from the term thermal acclimation to represent phenomena at the organism- and ecosystem-levels with different underlying mechanisms. Measurable physiological adjustments of the soil microbial biomass reflect shifts from colder- to warmer-adapted taxa. Hypothesized declines in the growth efficiency of soil microbial biomass under warming are controversial given limited data and a weak theoretical basis. I suggest that energy spilling (aka waste metabolism) is a more plausible mechanism for efficiency declines than the commonly invoked increase in maintenance-energy demands. Energy spilling has many fitness benefits for microbes and its response to climate warming is uncertain. Modeled responses of soil carbon to warming are sensitive to microbial growth efficiency, but declines in efficiency mitigate warming-induced carbon losses in microbial models and exacerbate them in conventional models. Both modeling structures assume that microbes regulate soil carbon turnover, highlighting the need for a third structure where microbes are not regulators. I conclude that microbial physiology must be considered if we are to have confidence in projected feedbacks between soil carbon stocks, atmospheric CO_2_, and climate change.

## INTRODUCTION

### CLIMATE-CARBON CYCLE FEEDBACKS

Respiration emits ~120 Pg C-CO_2_ per year from a terrestrial biosphere store of >2,000 Pg C to an atmospheric store of ~750 Pg C-CO_2_ (Steffen et al., 1998; Falkowski et al., 2000; Jobbágy and Jackson, 2000; Denman et al., 2007). This respiratory efflux is balanced annually by CO_2_-fixation by land plants (Steffen et al., 1998; Denman et al., 2007). This balance may be destabilized by climate warming because respiration rates respond more positively to increasing temperature than photosynthetic rates (Ise et al., 2010; Mahecha et al., 2010; Yvon-Durocher et al., 2010; Smith and Dukes, 2013). The net effect of this imbalance under warming is presumed to be a redistribution of organic carbon stored in the biosphere to carbon stored as CO_2 _ in the atmosphere (Denman et al., 2007). This redistribution might initiate a positive feedback (i.e., self-reinforcing) cycle, where elevated respiration rates enhance the rate of increase in atmospheric CO_2_ concentrations, which in turn warms climate, enhancing respiration and so on to cause runaway greenhouse warming. This presumed feedback is captured in the coupled climate-carbon cycle models used by the Intergovernmental Panel on Climate Change (IPCC), and leads to an additional, global mean annual warming of ~2°C by the year 2100 (Denman et al., 2007). In the IPCC models, the carbon lost from the biosphere to atmosphere derives from mineralization of soil organic matter (SOM), a carbon store to 3-m depth that is approximately triple the size of the atmospheric store (Jobbágy and Jackson, 2000) and so has huge potential to warm climate if converted to CO_2_.

**FIGURE 1:**
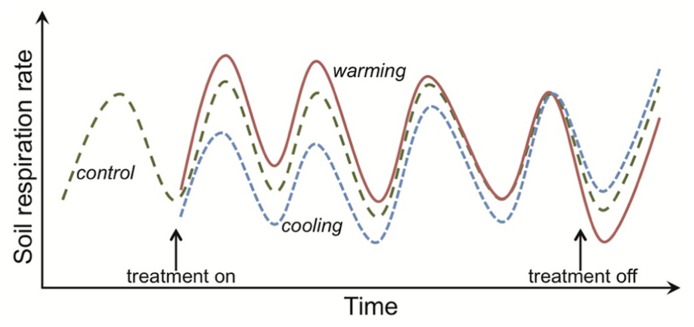
**Response of soil respiration to experimental warming or cooling across time in a mesic, temperate system.** The rates shown are not observations for any one experiment, but instead are intended to capture characteristic dynamics in respiration responses to warming. The hatched green line depicts respiration from control plots, where each unimodal cycle represents the expected increase in respiration rates across the growing season and then decline as plants senesce. Rates represent both autotrophic and heterotrophic respiration, and so higher rates in the growing season result from multiple mechanisms, including direct temperature effects on respiration and indirect effects such as higher plant-carbon supply to heterotrophic microbes. Similar dynamics would be expected for only heterotrophic respiration given that temperatures are higher during the growing season and the common assumption of a constant substrate supply, making it hard to disentangle the mechanisms underlying apparent thermal acclimation (see main text). Initiation of the warming treatment (e.g., a 5°C increase above ambient; depicted by the red solid line) stimulates soil respiration but this augmentation is ephemeral, with rates in the warming plots being equivalent to the controls by the fourth treatment year. This apparent thermal acclimation could arise through physiological adaptation of the organisms and/or changes in the environment, such as depletion of substrate that supports microbial activity. Both types of mechanism result in respiration rates in warmed plots that are lower than in controls if the treatment is discontinued. The opposite respiration response is observed for experimental cooling (blue dotted line).

Uncertainty about the strength of the positive feedback between warming, SOM mineralization and atmospheric CO_2_ concentrations (Melillo et al., 2002; Denman et al., 2007; Luo, 2007; Allison et al., 2010) has motivated the study of how SOM decomposition responds to elevated temperature. Much of the research involves investigating how temperature affects the activity (primarily respiration) of decomposers (Conant et al., 2011). A key question has been whether decomposer communities actively down-regulate their metabolism (i.e., acclimate) under sustained warming, and hence contribute to the diminishing effect over time of experimental warming on soil and ecosystem respiration rates (Oechel et al., 2000; Luo et al., 2001; Melillo et al., 2002; Bradford et al., 2008a; Reich, 2010; **Figure 1**). Numerical models demonstrate that physiological acclimation does not need to be invoked to explain the ephemeral augmentation of soil respiration in response to a fixed and sustained increase in temperature above ambient (Allison and Martiny, 2008; Kirschbaum, 2004; Eliasson et al., 2005; Knorr et al., 2005; Allison et al., 2010). This has prompted people to question why and by what mechanisms soil decomposer communities would down-regulate their physiological rates when “temperature limitations” are alleviated (e.g., Hartley et al., 2008). These seem fair questions because soil decomposers are poorly represented in the vast literature on how plants, animals and microorganisms physiologically adapt to temperature change (Crowther and Bradford, 2013). This poor representation is likely because it is difficult to study (a) cryptic organisms in an opaque environment and (b) organisms that are challenging to isolate and culture under laboratory conditions. These difficulties preclude soil decomposers from being subjected to the detailed physiological work on individual responses to temperature that is the hallmark of so many plant and animal studies (Hochachka and Somero, 2002; Atkin and Tjoelker, 2003).

The paucity of data on the physiological response of soil decomposer communities to warming is gradually being redressed (e.g., Bradford et al., 2008a; Bárcenas-Moreno et al., 2009; Brzostek and Finzi, 2011; German et al., 2012; Rousk et al., 2012; Crowther and Bradford, 2013; Tucker et al., 2013). Explicitly representing these physiological responses in the new class of microbial SOM models (e.g., Lawrence et al., 2009; Allison et al., 2010) predicts a short-lived increase in soil respiration under sustained warming (**Figure 1**). That is, the same respiration response as projected by the traditional SOM models, where decomposers are implicit in the model frameworks (Parton et al., 1988; Schimel, 2001; Eliasson et al., 2005; Bonan et al., 2013). Yet the projections for SOM stocks under warming contrast markedly between the microbial and traditional SOM models. The traditional models project large SOM stock losses, but the microbial models project little change in SOM stocks and hence no feedback to climate warming (Eliasson et al., 2005; Knorr et al., 2005; Kirschbaum, 2006; Allison et al., 2010).

### PURPOSE AND FRAMEWORK OF REVIEW

Explicitly representing microbes in SOM models, and then embedding them in land-ecosystem and hence Earth System Models (ESMs), faces a number of challenges (Schimel, 2001; Bradford and Fierer, 2012; Todd-Brown et al., 2012; Treseder et al., 2012). One of these challenges is establishing a common conceptual framework through which researchers in a diverse set of fields, including physiology, microbial ecology and ecosystem ecology, can productively interact. I aim to help provide this common framework by:

Section 2 – Clarifying the meaning of the terms thermal acclimation and adaptation

Section 3 – Describing mechanisms underlying soil respiration responses to warming that are independent of direct temperature effects on microbial physiology

Section 4 – Reviewing direct responses of microbial physiology to warming

Section 5 – Discussing theoretical challenges to incorporating microbes into SOM, ecosystem and Earth System Models.

Much of my review focuses on respiratory processes because (a) at the ecosystem-level for soil responses to warming the literature focuses primarily on respiration; and (b) a substantial proportion of physiological work on thermal adaptation has focused on respiration. The caveat, however, is that thermal adaptation refers to a suite of phenomena (Hazel, 1995; Hochachka and Somero, 2002; Angilletta, 2009) and I devote considerable discussion to microbial growth because it has marked potential to affect how global and local SOM stocks respond to warming.

**FIGURE 2:**
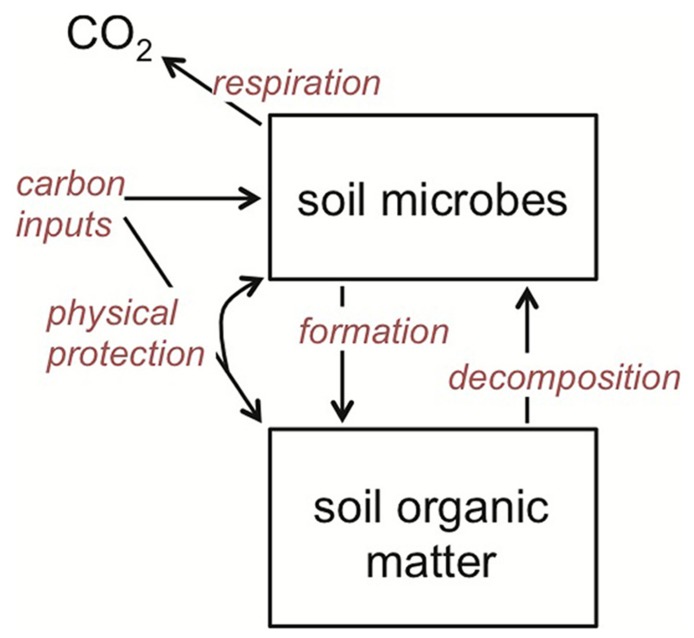
**Theoretical framework for soil organic matter (SOM) dynamics, emphasizing the central role that soil microbes play in both SOM decomposition and formation.** The rate of all of the processes (red italics) is temperature dependent, and hence stock sizes of SOM will be dependent on how warming affects the rate of each relative to one another.

I concentrate on soil microorganisms that decompose organic matter using extracellular enzymes and/or assimilate low molecular weight organic compounds from the soil environment. These organisms include free-living, heterotrophic microbes in the litter, bulk soil and rhizosphere, as well as those that are plant mutualists, such as ectomycorrhizal fungi. What they have in common is that together they are the primary biotic agents in terrestrial systems regulating the breakdown of organic matter and its eventual mineralization and hence return to the atmosphere as CO_2_ (**Figure 2**). These organisms are also primary agents of SOM formation (Grandy and Neff, 2008; Schmidt et al., 2011; Miltner et al., 2012; Bradford et al., 2013; Clemmensen et al., 2013), suggesting that it is the balance of their changing catabolic and anabolic activities under warming that together determine SOM stocks (**Figure 2**).

## WHAT IS THERMAL ACCLIMATION?

### DEFINITIONS

The scientific literature is burdened with a variety of uses for the same term. The confusion created hinders discourse across disciplines, presenting an obstacle to the interdisciplinary science demanded by environmental problem solving (National_Research_Council, 2009). The terms thermal acclimation, acclimatization and adaptation are all variously used to represent direct and indirect effects of temperature on soil microbial activity. More than a half-century ago, Bullock (1955) decided not to perpetuate the multifarious uses of these terms and, following his lead, I define here the terms I use but do not expect others necessarily to adopt them. I follow Hochachka and Somero (2002) by using ‘thermal adaptation’ as an integrative term that captures *direct* organism responses to temperature across immediate to multi-generational time-scales that manifest as physiological change. This then permits me to discuss thermal adaptation in soil communities without pretending to know the precise mechanisms underlying the adaptive response, because we simply do not yet know which mechanisms contribute most to thermal adaptation in soil microbial activity. These mechanisms operate across three distinct timescales.

The initial adaptations involve changes in active biochemical systems within cells, such as the availability of intracellular carbohydrates whose depletion limits cellular respiration rates (Tjoelker et al., 2008). Over days to a few weeks intermediate timescale adaptations occur, which modify preexisting biochemical systems through synthesis of new or different quantities of cellular machinery (e.g., enzymes). Such intermediate timescale physiological adjustments within individuals are commonly referred to as acclimation or acclimatization (Hochachka and Somero, 2002). Longer timescale adaptations involve evolutionary change but can span few to many generations. For example, species/genotype turnover might occur across few generations, where temperature acts on existing genetic variation among organisms to select those best adapted to grow at the new environmental temperature. In contrast, selection of beneficial *de novo* mutations could take many generations (Hochachka and Somero, 2002). Later in this review I present arguments that adaptations that influence the activities of soil decomposer communities at ecosystem-scales at management-relevant timescales (i.e., <30 years), in response to warming at a location, likely arise through species/genotype turnover.

I use the term “apparent thermal acclimation” (e.g., as in Tucker et al., 2013) to connote an ephemeral augmentation in soil and ecosystem respiration rates to prolonged warming that result from indirect effects of temperature on microbial activity such as, for example, reductions in SOM or moisture availability. This definition and the one I use for thermal adaptation are then consistent with expected responses of respiration to prolonged warming at both organism- and ecosystem-levels. That is, for an initial increase in respiration under warming to diminish or for recovery of an initial decrease in respiration under cooling (**Figure 1**).

### MEASURING THERMAL ADAPTATION OF RESPIRATION

Thermal adaptation of respiration involves dampening in the response of mass-specific respiration rates to temperature change. Mass-specific respiration (*R*_mass_) rates are calculated as respiration per unit biomass, making the measurement of individual, population and/or community biomass essential for calculating and discussing thermal adaptation of respiration.

**FIGURE 3:**
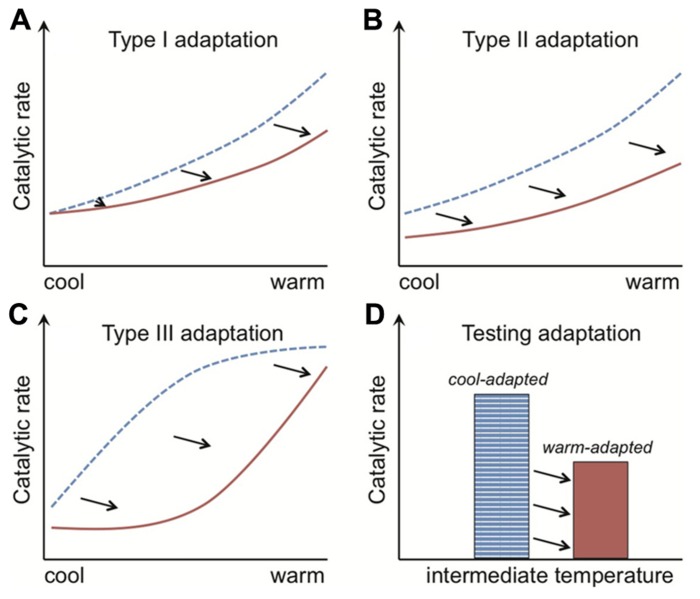
**The rate of enzyme-mediated reactions are shifted right along the temperature axis for organisms adapted to warmer conditions.** The catalytic rates of both cool- and warm-adapted enzymes (blue dotted and red solid lines, respectively) increase with temperature. Their temperature response pattern, however, differs. With type I adaptation **(A)** the temperature sensitivity (i.e., Q_10_) of warm-adapted enzymes is lower, and with type II adaptation **(B)** this sensitivity is unchanged but the absolute catalytic rates are consistently lower across the temperature gradient. Type III adaptation **(C)** represents a mix of type I and II adaptation and is associated with shifts from cold- to warm-adapted communities, where adaptation leads to separation of the temperature response into discrete, bell-shaped curves (see **Figure 5**). The commonality in pattern across the type I to III responses is that the temperature optima of warm-adapted enzymes, individuals, populations or communities is shifted right of the cold-adapted lines. The black arrows depict this “right shift.” The classical test for thermal adaptation **(D)** involves measuring process rates at an intermediate temperature (i.e., between cool and warm), where types I–III adaptation would always produce lower rates for the warm-adapted communities, assuming variables such as substrate supply are non-limiting and rates are standardized by organism biomass.

Adaptation of *R*_mass_ to warming is exhibited through a dampening of Q_10_ (type I adaptation) and/or a change in absolute *R*_mass_ rates at any one temperature (type II adaptation; Atkin and Tjoelker, 2003). The metric “Q_10_” is commonly used to estimate temperature sensitivity, where for example a value of 2 means that respiration rates double per 10°C rise. Type I and II adaptation patterns (**Figures 3A,B**) are achieved at the cell level through, for example, changes in the inherent properties of enzymes that determine the temperature sensitivity or absolute magnitude of their catalytic rates, respectively. Type II adaptation dampens respiration responses to a sustained temperature change without adjustment of temperature sensitivity. Bradford et al. (2008a) define a third class of adaptation (type III), where a shift from a cold- to warm-adapted community or vice versa leads to a fundamental change in the temperature response of *R*_mass_ (**Figure 3C**). Such community shifts have the potential to generate seemingly paradoxical Q_10_ values; where over the same temperature range warm-adapted communities have elevated (as opposed to dampened) Q_10_ values. These elevated Q_10_ values could arise because measured Q_10_ typically decreases across the temperature range over which respiration is active (Davidson and Janssens, 2006), and so a warm- vs. cool-adapted community falls at an earlier part of its active range at intermediate temperatures (**Figure 3C**). I am not aware of this phenomenon of elevated Q_10_ values being shown for soil microbial respiration, but the same mechanism might explain why soil communities adapted to warmer temperatures have higher Q_10_ values for growth (Rousk et al., 2012). The commonality in pattern across the type I to III *R*_mass_ responses is that the temperature optima for respiration of warm-adapted enzymes, individuals, populations or communities are shifted right of those of cold-adapted organisms (Hall et al., 2008; Tjoelker et al., 2008; Bradford et al., 2010).

The “right shift” in temperature optima sets up the classical test for thermal adaptation of respiration (**Figure 3D**). This classical test relies on the fact that cold-adapted organisms should have higher *R*_mass_ rates, at intermediate temperatures, than warm-adapted organisms because the temperature optimum for the latter has been shifted right (**Figure 3**). The test needs to be performed under conditions where other factors do not limit respiration. For example, for the soil microbial community one should ensure that soil moisture and substrate availability are non-limiting (Bradford et al., 2008a, 2010). Both moisture and substrate limitation restrict respiration responses to temperature (Gu et al., 2004; Bengtson and Bengtsson, 2007; Almagro et al., 2009; Davidson et al., 2012; Suseela et al., 2012; Tucker et al., 2013), and substrate limitation at least in part explains diminishing soil respiration rates under sustained experimental warming (Hartley et al., 2007; Bradford et al., 2008a; Tucker et al., 2013). Tests for thermal adaptation must account also for differences in microbial biomass because higher biomass usually means higher respiration (Waldrop et al., 2000; Allison et al., 2010), which explains why tests for thermal adaptation of respiration must measure *R*_mass_.

### WHY IS THERMAL ADAPTATION IN SOIL DECOMPOSER COMMUNITIES STILL DEBATED?

When expressed as *R*_mass_, both field and laboratory warming experiments have shown thermal adaptation of soil respiration (Bradford et al., 2008a, 2010; but see Bradford et al., 2009; Hartley et al., 2009). Yet purported empirical tests of thermal adaptation of soil respiration rarely control for differences in microbial biomass and/or substrate availability (e.g., Hartley et al., 2007, 2008; Vicca et al., 2009). In modeling studies, both Knorr et al. (2005) and Kirschbaum (2004) concluded that adaptation in soil microbes was not required to explain apparent thermal acclimation in soil respiration because substrate depletion (see Indirect Effects of Temperature on Microbial Activity) generated the respiration response. They could not, however, falsify the hypothesis that thermal adaptation might also explain apparent thermal acclimation because they did not model thermal adaptation as a competing mechanism. This rule in mathematical modeling is often quoted as “pattern does not beget process,” and cautions against accepting as proof of mechanism a model that recreates the observed pattern (Warren et al., 2011b). Indeed, when Allison et al. (2010) modeled both adaptation and substrate depletion, they found both were plausible mechanisms explaining apparent thermal acclimation in soil respiration. The Knorr et al. (2005) and Kirschbaum (2004) studies, however, have >700 citations between them, suggesting they were influential in proliferating the idea that heterotrophic soil microbes might not adapt to warmer temperatures, even though we expect adaptation in other organisms that drive terrestrial carbon cycling (Reich, 2010).

Incorporation of knowledge from other disciplines and direct tests of adaptation seem to underlie recent advances in evaluating how adaptive microbial responses under warming affect SOM dynamics. These advances go beyond respiratory responses and assess other physiological parameters, such as growth efficiencies and extracellular enzyme activities (German et al., 2012; Manzoni et al., 2012b; Wallenstein et al., 2012; Frey et al., 2013; Tucker et al., 2013). These investigations are finding evidence for adaptation and, as a consequence, generate different expectations for how warming will influence SOM stocks (e.g., accelerated loss vs. protection of stocks; Allison et al., 2010; German et al., 2012; Frey et al., 2013; Tucker et al., 2013). Nevertheless, the extent to which indirect vs. direct temperature effects drive SOM dynamics under warming is largely untested (see Rousk et al., 2012), although the effect types co-occur in field experiments (Bradford et al., 2008a). To provide sufficient space to review and discuss direct temperature effects, I only briefly cover indirect effects. This brevity should not be misinterpreted: indirect effects undoubtedly have a major influence on SOM responses to warming and a synthesis of these effects seems warranted.

## INDIRECT EFFECTS OF TEMPERATURE ON MICROBIAL ACTIVITY

Apparent thermal acclimation of soil respiration can arise through multiple processes because biological CO_2_ effluxes represent the cumulative activity of microbes, plants and animals (Boone et al., 1998; Ostle et al., 2009). Conventional SOM models assume that indirect effects provide the sole explanation for longer-term respiration and SOM responses to sustained warming (Kirschbaum, 2004; Eliasson et al., 2005; Knorr et al., 2005) and make no predictions as to how microbial community composition and biomass are affected. For example, substrate depletion is the classic indirect mechanism by which soil respiration is “down regulated” under prolonged warming (**Figure 1**). The mechanism has both observational and experimental support. For example, labile carbon availability to the microbial biomass is lower in experimentally warmed soils (Hartley et al., 2007; Bradford et al., 2008a; Curiel Yuste et al., 2010). Further, seasonal patterns in soil respiration responses to temperature are strongly dependent on substrate availability, with temperature having minimal effects on respiration rates at times of the year when substrate is depleted, and strong effects when substrate supply is abundant (Gu et al., 2004; Bengtson and Bengtsson, 2007). This all makes perfect sense: if an ectothermic heterotroph cannot get much to eat, and has depleted any internal stores, then its *R*_mass_ rate will decrease under otherwise constant environmental conditions.

**FIGURE 4:**
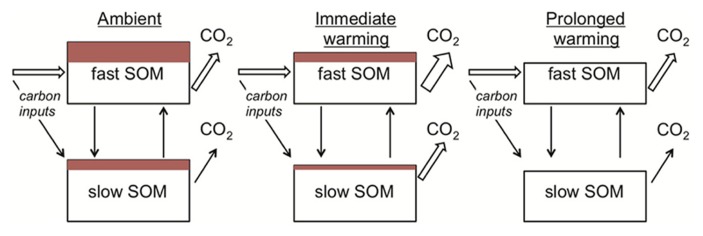
**The mechanistic basis for apparent thermal acclimation of soil respiration rates as represented in conventional soil organic matter (SOM) models.** Under ambient conditions (left) a proportion of the SOM in both fast and slow pools is vulnerable to loss if temperatures increase (depicted as red-filled rectangles). On warming (middle) respiration rates increase because the rate of SOM turnover is positively related to temperature and a fixed proportion of this turnover is lost as CO_2_. The red-filled rectangles decrease because carbon losses in respiration are greater than plant-carbon inputs to the soil. With prolonged warming (right) all of the vulnerable SOM is lost as CO_2_, depicted as loss of the red-filled rectangles and reductions in the SOM pool sizes. Respiration rates under ambient and prolonged warming conditions are the same because carbon input rates equal loss rates under steady-state conditions, creating “apparent” thermal acclimation (see Definitions for definition).

Apparent thermal acclimation in the respiration responses of conventional SOM models also appears consistent with substrate depletion. When warming is imposed in conventional SOM models, the turnover rate of the SOM pools increases. The implicit biological assumption is that temperature constraints on microbial activity are relaxed under warming. As the modeled SOM pools turnover, a constant fraction of the carbon is lost as CO_2_. Hence faster turnover is associated with greater losses of CO_2_ per unit time (**Figure 4**). This dynamic causes the initial stimulation of soil respiration under warming (**Figure 1**). Gradually, however, the SOM pool of interest decreases in size (i.e., substrate depletion) and, as it does so, there is a proportional decline in respiration from this pool. At the new steady state (i.e., conditions under which SOM pool sizes are constant), soil respiration rates equal carbon input rates to the soil (**Figure 4**). Conventional SOM modeling studies have kept carbon input rates equal under ambient and warmed conditions (Kirschbaum, 2004; Knorr et al., 2005). Hence, under this assumption respiration rates at steady state from ambient and warmer conditions are identical (**Figures 1, 4**), although faster cycling SOM pools are smaller in warmed soils (**Figure 4**). These effects of warming appear consistent with our understanding of limiting factors on microbes in mineral soils: SOM decomposers typically exist in an environment where substrate is limiting (Schimel and Weintraub, 2003).

Although depletion of SOM-substrates has received most attention as the mechanism underlying indirect effects of warming on microbial activity, temperature also influences numerous other processes that influence substrate availability to microbes and so would be expected to modify microbial activity in warming soils. For example, substrate supply rates are a critical control on SOM stocks and turnover, and the temperature response of respiration (Cheng et al., 1996; Fontaine and Barot, 2005; Bradford et al., 2008b; Gershenson et al., 2009; Kuzyakov, 2010; Dorrepaal et al., 2013). Substrate supply is affected by plant carbon input rates, which have been shown to both decrease and increase under warming (Uselman et al., 2000; Ise et al., 2010; Yin et al., 2013). Similarly divergent responses have been shown for fine roots, which are important not only for rhizodeposition but as carbon substrates themselves (Pregitzer et al., 2000; Rinnan et al., 2008; Melillo et al., 2011; Sistla et al., 2013). Substrate supply within the soil will also be altered if warming affects soil moisture because water availability affects the rate at which enzymes, substrates and/or products of degradation diffuse between microbes and their immediate environment (Xu and Saiers, 2010; Davidson et al., 2012; Manzoni et al., 2012a). For example, if warming dries a soil then diffusion rates may decrease, reducing substrate availability to microbes. Temperature also controls the rate at which substrates sorb and desorb from organo-mineral surfaces (Conant et al., 2011), and hence become available to microbes. Further, temperature may decrease overall SOM substrate quality because labile substrates are depleted faster than more recalcitrant substrates, decreasing both the availability and quality of SOM-substrates (Davidson and Janssens, 2006).

Warming effects on substrate availability – through the mechanisms outlined above – in addition to other warming-induced effects on soil variables such as nitrogen availability (Rustad et al., 2001; Melillo et al., 2011), seem likely to lead to changes in microbial decomposer communities that in turn influence soil respiration rates under warming. For example, substrate limitation might shift enzyme expression toward higher affinity enzymes (Steinweg et al., 2008), where the trade-off is a reduction in maximum catalytic rates. Such a shift in enzyme expression would favor a slower growing microbial biomass, and lower respiration rates, recreating expected reductions in microbial biomass and respiration under sustained warming. Overall, then, a broad array of indirect mechanisms under warming likely affect microbial physiology and community composition, and these indirect effects likely co-occur with the direct effects of warming that are discussed next.

## DIRECT EFFECTS OF TEMPERATURE ON MICROBIAL ACTIVITY

Temperature is a fundamental determinant of the distribution and abundance of organisms across time and space (Angilletta, 2009). Organisms occupy different thermal niches because of their physiological tolerances and because temperature modulates the strength of both positive and negative biotic interactions (e.g., Warren et al., 2011a; A’Bear et al., 2012). These individual responses and biotic interactions translate to differences in fitness across genotypes and species. As a result populations subdivide into thermal ecotypes and communities differ in composition as species sort based on environmental temperature (Porankiewicz et al., 1998; Hall et al., 2008, 2010; Wallenstein and Hall, 2012; Garcia-Pichel et al., 2013). Not surprisingly then, experimental and observational studies demonstrate that temperature drives microevolution and speciation (Leroi et al., 1994; Turner et al., 1996; Cooper et al., 2001; Angilletta, 2009). Warming should then directly affect microbial community physiology, biomass and composition (Zogg et al., 1997; Bardgett et al., 1999; Frey et al., 2008, 2013; Bradford et al., 2010; German et al., 2012; Rousk et al., 2012). Investigations of adaptive responses to warming of soil decomposer communities, however, have primarily focused on community-level respiration and growth (Ranneklev and Bååth, 2001; Bradford et al., 2008a, 2010; Bárcenas-Moreno et al., 2009; Rinnan et al., 2009; Rousk and Bååth, 2011; Rousk et al., 2012). I review these two processes first, before discussing how warming might affect biotic interactions, such as microbivory, that could mitigate or exacerbate microbial respiration and growth responses to temperature change.

A focus on the aggregate, or community-level, response of microbial respiration and/or growth means that adaptation might manifest through multiple mechanisms, ranging from shifts in individual physiology to changes in species composition. That multiple mechanisms are operating obscures our ability to ascribe specific causation as to why we observe thermal adaptation. For example, *R*_mass_ rates of warm-adapted individuals are expected to be lower than those of cool-adapted individuals at intermediate temperatures, but this result could arise through a change in enzyme expression and/or changes in cell membrane structure. These individual responses might translate to the community *R*_mass_ response, but equally there could be turnover in community composition toward warm-adapted genotypes or species (Bradford et al., 2008a, 2010; Hartley et al., 2008; Wallenstein and Hall, 2012). In their work on biochemical adaptation, Hochachka and Somero (2002) cautioned that the physiological mechanism explaining adaptation in the rate of a process could be obscured when working at the level of an organ within an individual animal. They advised working at the intracellular level to explain causation. Such work is no doubt required for soil decomposer organisms but we are far from such a reality. Can any of us even state categorically what the most important microbial taxa are for decomposing SOM? We just do not know which study species to choose.

If we do observe a change in microbial community composition under warming, relating such shifts to changes in soil functioning, let alone the pattern of thermal adaptation, is still a major challenge for soil microbial ecologists (Allison and Martiny, 2008; Bradford and Fierer, 2012; Wallenstein and Hall, 2012). Isolation and pure-culture offer an approach to look at physiological responses to warming that might be expected to reflect general responses and hence universal constraints on organisms (Lennon and Jones, 2011). However, linking these single species back to the aggregate responses of multi-species communities is challenging. The best approach seems to be to recognize that multiple processes might underlie thermal adaptation responses. We must then investigate each mechanism, to determine which contribute most to ecosystem-level carbon cycling responses to climate change. In the subsections below I therefore explore mechanisms that span from the individual- to community-level, and do not pretend to know which matter most for explaining warming effects on carbon cycling at the ecosystem-level.

### RESPIRATION

#### Trade-offs in enzyme structure and function

**FIGURE 5:**
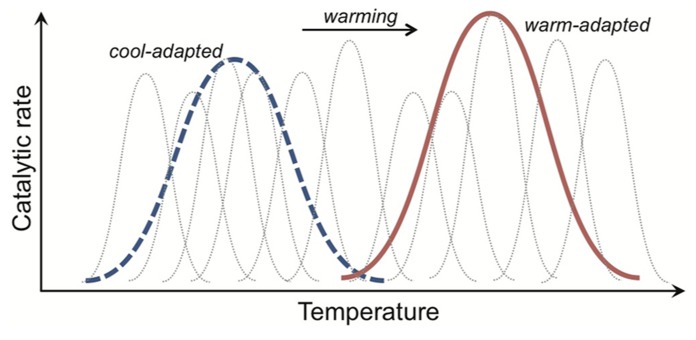
**Catalytic rate responses to temperature, caused by the evolutionary trade-off between enzyme structure and function, giving rise to different distributions of isoenzymes in cool- and warm-adapted organisms.** An isoenzyme (gray dotted curves) is an enzyme with the same function but a different structure. In any one organism, population or community we expect a different set of isoenzymes to be expressed at different temperatures. This is because enzyme structure usually only promotes efficient substrate-binding and product-release across a narrow temperature window. As such, the aggregate activity of a cool- or warm-adapted organism, population or community (depicted as thick blue dotted and red solid lines for cool- and warm-adapted) results from the activities of a family of isoenzymes expressed across the environmental temperature range.

Enzyme-mediated reactions are generally temperature sensitive. An increase in temperature accelerates reaction rates in the short-term, when all other variables are non-limiting (e.g., enzyme and substrate availabilities). At high temperatures proteins denature and so enzyme function and hence reaction rates drop precipitously. Long-before reaching denaturing temperatures, however, the rate of increase in an enzyme-mediated reaction decreases. Fundamental trade-offs in enzyme structure and function underlie this deceleration. Essentially, enzymes need to be folded into certain three-dimensional shapes (i.e., conformations) to bind a substrate and other shapes to release the product. The rate at which they change shape theoretically controls the speed of the reaction the enzyme catalyzes. At lower temperatures, these shape changes are faster for more flexible enzyme structures. At warmer temperatures, however, a flexible enzyme spends less time in shapes that bind substrate, which decreases the affinity of the enzyme for substrate and hence reduces the relative rise in reaction rate with increasing temperature (**Figure 5**). Temperature then selects (in the Darwinian sense) for more flexible enzymes when it is cooler and for less flexible enzymes when it is warmer.

A less flexible enzyme can maintain binding conformations for a greater proportion of time at warmer temperatures, and so “outcompete” a cool-adapted enzyme for substrate. As such, in any one organism, population or community, we expect a different set of isoenzymes to be expressed at different temperatures (**Figure 5**). An isoenzyme (aka isozyme) is an enzyme with the same function but a different structure. In environments where temperature varies markedly in the short-term, such as the day-night cycle during the growing season in temperate forest, a broad suite of isoenzymes might be expressed but the relative contribution to catalysis of any one isoenzyme will change with the daily temperature cycle (**Figure 5**). Across broad latitudinal gradients, however, catabolic responses for communities from lower latitudes appear shifted right along the temperature axis, consistent with expected trade-offs (Balser and Wixon, 2009; German et al., 2012).

#### Will trade-offs in respiratory enzymes affect ecosystem processes?

Most of the biochemical adaptation work on trade-offs in flexibility under temperature has been conducted with enzymes involved in the metabolic pathways that comprise cellular respiration. The CO_2_ of aerobic respiration is generated as an intracellular product. Ecosystem respiration fluxes therefore result from reactions catalyzed by intracellular enzymes. It would be a mistake, however, to infer that physiological adjustments in *R*_mass_ rates in warm vs. cold-adapted microbial communities (**Figure 3**) necessarily underlie apparent thermal acclimation of soil respiration. For example, Schimel and Schaeffer (2012) posited that microbial communities only control ecosystem processes when two conditions hold: (a) organisms differ in their functional traits and (b) the biological process is the rate-limiting step in the reaction sequence.

Condition (a) should hold for the traits of cold- and warm-adapted communities, given the fundamental evolutionary trade-offs between enzyme structure and function across temperature. That is, *R*_mass_ rates will differ if measured under standard conditions (**Figure 3**) because of physiological adjustments that arise through individual or community responses. There is some evidence for these shifts in functional traits for soil microbial communities and for laboratory-grown heterotrophic microbes (Balser and Wixon, 2009; Bradford et al., 2010; German et al., 2012; Crowther and Bradford, 2013). If we look more broadly, we find evidence for the trade-off in mycorrhizal fungi (Heinemeyer et al., 2006; Malcolm et al., 2008) and for heterotrophic microbes and their communities across a range of other systems, where there are distinct latitudinal and seasonal patterns in *R*_mass_ as a consequence of environmental temperature (Porankiewicz et al., 1998; Lange and Green, 2005; Clarke, 2006; Hall and Cotner, 2007; Tjoelker et al., 2008). Given the theoretical and empirical evidence, the physiological function of heterotrophic soil microbial communities must adapt to warming.

For functional trait differences to influence soil respiration responses to warming requires Schimel and Schaeffer’s (2012) condition (b) to hold. That is, that microbial activity is the rate-limiting step in SOM decomposition. Yet they argue this is not the case for mineral soils. Instead, they suggest that physical protection of SOM (e.g., sorption) regulates the breakdown rate of SOM. We should therefore expect temperature-induced changes in intracellular microbial physiology not to scale to soil and ecosystem respiration rates. Schimel and Schaeffer (2012) argue that formation of SOM may, however, be conditional on microbial community composition because organisms differ in their biochemical make-up (e.g., the amount of lipids) and compounds differ in the extent to which they are physically protected from decay. If true, SOM decomposition rates might be independent of physiological adjustments but SOM stock sizes will be dependent on the physiology of the overall microbial community. Specifically, stock sizes are a product of both losses (i.e., decomposition) and inputs (i.e., formation). Understanding soil respiration responses to warming is then likely a poor indicator of SOM stock responses (Conant et al., 2011; Hamdi et al., 2013), meaning that SOM stocks and turnover must be measured directly to understand climate-carbon cycle feedbacks.

Warming does influence the biochemical composition of microorganisms and hence could affect SOM formation rates. For example, as with enzymes, there are trade-offs between the structure and function of lipids in cell membranes (Hazel and Williams, 1990; Hazel, 1995). These trade-offs influence cell membrane permeability and translate to lower *R*_mass_ values for warm- vs. cold-adapted organisms when testing for thermal adaptation (Hochachka and Somero, 2002). There is no direct evidence of warming-induced changes in cell membrane structure for soil decomposers, but the trade-offs have been shown for aquatic microbial heterotrophs (Hall et al., 2010). The potential for shifts in the chemical composition of soil microbes to affect SOM formation rates, however, remains to be tested. Given that stable SOM in well-drained mineral soils appears to be largely composed of microbial-derived products (Lundberg et al., 2001; Grandy and Neff, 2008), this possibility seems a research priority.

The idea that microbial activity in mineral soils does not regulate SOM decomposition rates is controversial (Kemmitt et al., 2008; Kuzyakov et al., 2009; Paterson, 2009; Schimel and Schaeffer, 2012; Thiessen et al., 2013). It does seem certain, however, that microbial community composition affects the breakdown and mineralization rates of leaf litter (Strickland et al., 2009; Wallenstein et al., 2010, 2012; Keiser et al., 2011; Schimel and Schaeffer, 2012). In some systems litter breakdown (foliar and woody) can account for a substantial fraction of ecosystem respiration (Wu et al., 2005; Weedon et al., 2009), and so in these systems we might expect physiological shifts in microbial communities under warming to translate to the ecosystem-level. At the very least, for mineral soils, such shifts will influence nutrient cycling because they regulate litter decomposition rates, and so might indirectly affect ecosystem-level carbon fluxes through influences on plant growth. In organic soils the SOM is not protected by organo-mineral interactions, and so this presumably also makes its breakdown sensitive to microbial physiology. As high-latitude systems warm, constraints on microbial activity such as frozen water may be relaxed, making huge stocks of SOM in organic permafrost soils vulnerable to mineralization. Physiological responses of microbes to warming will then influence climate-carbon cycle feedbacks if microbial activity is a rate-limiting step in the breakdown of organic soil carbon stocks.

#### Extracellular enzymes

If microbial activity does regulate how temperature affects the breakdown of SOM stores, the accepted wisdom is that extracellular (not intracellular) biological processes provide the rate-limiting step (Allison et al., 2011; Wallenstein and Hall, 2012). Specifically, soil microbes catalyze the breakdown of SOM using extracellular enzymes, where the dissolved, low molecular weight products can be assimilated. The enzymes involved in assimilation, intracellular metabolism, and extracellular degradation should all be under the same evolutionary pressure to generate the trade-off between structure and function. Enzymes involved in assimilation of dissolved compounds from the soil environment have not been investigated for this trade-off, but the aggregate activity of classes (e.g., cellulases) of extracellular enzymes expressed by soil decomposer communities do respond to seasonal, latitudinal and experimental warming in a manner consistent with thermal adaptation (Fenner et al., 2005; Wallenstein et al., 2009; Brzostek and Finzi, 2011, 2012; Brzostek et al., 2012; German et al., 2012; Stone et al., 2012). Thermal adaptation in extracellular enzymes could affect warming responses of ecosystem and soil respiration if they provide a rate-limiting step for the acquisition of substrate by the soil microbial community, which in turn controls how much substrate microbes have available for respiration. The classical test for thermal adaptation of *R*_mass_ rates (**Figure 3**), however, would not detect thermal adaptation of extracellular enzymes because substrate is supplied in a form not requiring decay prior to assimilation. The test then only examines warming-induced shifts in cellular physiology, such as membrane structure and isoenzyme expression of assimilatory and intracellular enzymes.

### GROWTH

Microbial growth is likely much more important than respiration for determining climate-carbon cycle feedbacks. Colonization rates and hence the breakdown of new resources are a function of growth rates, extracellular enzyme production is tied to biomass production, and so are SOM formation rates (Waldrop et al., 2000; Rousk and Bååth, 2011; Schmidt et al., 2011; Bradford et al., 2013; Cotrufo et al., 2013; Thiessen et al., 2013). Microbial growth efficiency (MGE; aka carbon use efficiency) was the physiological parameter in the microbial SOM model of Allison et al. (2010) to which SOM stocks were most sensitive. Growth efficiency is broadly defined as the proportion of assimilated substrate allocated to growth vs. other fates such as respiration (Brant et al., 2006; Thiet et al., 2006; Frey et al., 2013). Under model scenarios where efficiencies declined in a constant linear fashion with increasing temperature, Allison et al. (2010) demonstrated that associated decreases in microbial biomass and hence extracellular enzyme activities meant that SOM stocks were protected from loss. Understanding how MGEs respond to temperature in the shorter- and longer-term is a research priority if we are to project reliably climate-carbon cycle feedbacks.

#### Microbial growth efficiency

That MGEs decline as environmental temperature increases is controversial. For heterotrophic microbial communities in aquatic systems, debate has raged as to whether substrate quality alone vs. temperature explains differences in growth efficiencies (del Giorgio and Cole, 1998; Rivkin and Legendre, 2001; Apple et al., 2006; Apple and del Giorgio, 2007; López-Urrutia and Morán, 2007). The idea that substrate quality matters is not controversial. More chemically recalcitrant substrates require greater energy investment to breakdown, reducing net energy gain and hence leaving less energy available for growth (Fierer et al., 2005; Davidson and Janssens, 2006; Craine et al., 2010). The mechanism by which increasing temperature reduces efficiencies is often thought to depend on maintenance energy costs being higher as temperature rises (Manzoni et al., 2012b). Greater maintenance costs then reduce the proportion of energy acquired that is available to growth. The two maintenance activities requiring most energy are likely protein synthesis and the maintenance of ionic gradients across membranes (Clarke and Fraser, 2004). The metabolic costs of maintaining these two processes, for an individual or community, immediately increase with warming because proteins (including enzymes) are less stable and membranes more permeable. These physiological consequences heighten ATP demand, driving respiration, and hence for a fixed substrate intake rate reduce the energy remaining for growth. In the intermediate-term, evolutionary trade-offs (see Respiration) suggest that isoenzymes and membranes will shift toward structures that are more warm-adapted. These shifts should explain thermal adaptation of MGEs. The empirical evidence in soil decomposer communities for shifts in efficiency with sustained warming is, however, limited to a single study and was observed for only one of four tested substrates (Frey et al., 2013).

Original observations that MGEs in soils declined with temperature were confounded by the complexity of substrates on which the microbial biomass was growing (Devêvre and Horwath, 2000; van Ginkel et al., 2000; Pietikäinen et al., 2005). Increasing temperatures permitted the microbes to use more chemically recalcitrant substrates, which have lower efficiencies. Frey et al.’s (2013) observation that MGEs declined with increasing temperature only for substrates requiring extracellular enzyme decay, helped resolve apparently conflicting results that MGEs were temperature insensitive (for glucose, Dijkstra et al., 2011) vs. sensitive (for cellobiose which requires degradation prior to assimilation, Steinweg et al., 2008). When MGEs of whole communities are temperature sensitive, they decline linearly or curvi-linearly with increasing temperature (Devêvre and Horwath, 2000; van Ginkel et al., 2000; Steinweg et al., 2008; Frey et al., 2013). In contrast, efficiencies are distinctly unimodal for isolates of free-living microbial heterotrophs, that produce extracellular enzymes for substrate decay, from both soils and other environments (Russell, 2007; Crowther and Bradford, 2013). Specifically, there appears to be an optimum MGE that matches the ambient temperature regime from the organism’s environment, which declines at cooler and warmer temperatures than this optimum. Growth efficiencies for individuals and communities should, like *R*_mass_, then conform to a suite of unimodal response curves (**Figure 5**). Why are individual growth efficiencies unimodal and those for soil communities unresponsive or only negatively affected by warming?

There is no clear answer as to why MGEs of soil communities are temperature insensitive (e.g., for glucose) or, when sensitive (e.g., for phenol), respond only negatively to temperature. It seems that our understanding of how MGEs respond to warming is woefully inadequate. In short, the theoretical basis is a physiological quagmire, arising from the complexity and associated unknowns of metabolism across all forms of life. For example, why do MGEs of whole communities decline with increasing temperature when using substrates that require extracellular decay (Steinweg et al., 2008; Frey et al., 2013)? There is weak support for the idea that declines occur because maintenance energy costs increase with temperature, leaving less for growth, albeit this explanation is commonly invoked in soil and ecosystem ecology (Clarke and Fraser, 2004; Clarke, 2006; Russell, 2007; Allison et al., 2010; Manzoni et al., 2012b). If maintenance costs do increase at the expense of growth, then catabolic and anabolic energy demands must be uncoupled, with more energy diverted to the former. These energy demands certainly do become uncoupled, with efficiencies declining at temperatures both above and below the optimum for growth in individuals (Angilletta, 2009; Crowther and Bradford, 2013). Yet higher maintenance energies only account for a small proportion of the elevated catabolic demand and there are even arguments that higher maintenance costs do not uncouple anabolic and catabolic processes.

**FIGURE 6:**
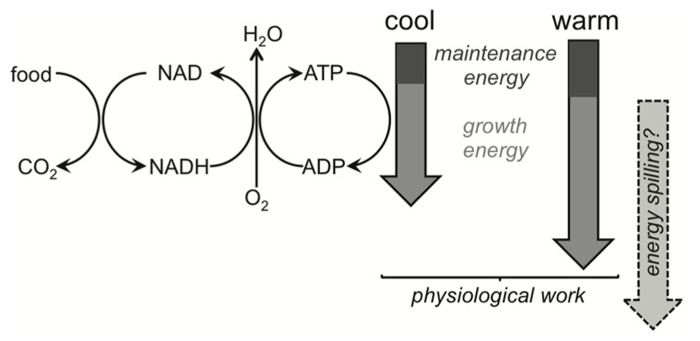
**Simplified schematic of metabolism, highlighting ATP-supply being driven by the demand of physiological work and/or energy spilling.** Organisms catabolize substrates to provide energy demanded by maintenance and growth. Catabolism and anabolism are then coupled under this metabolic scheme. Decreases in microbial growth efficiency (MGE) are expected under warming, and the commonly cited mechanism involves maintenance energy demands responding more strongly to warming than growth energy demands. The theoretical basis for this expectation remains to be demonstrated and MGEs in field soils can be invariant to temperature. Maintenance demands more likely increase proportionally with growth demands (shown by the cool and warm block arrows), and if temperature accelerates growth then maintenance costs might even become proportionally smaller, increasing MGEs. Energy spilling is an alternative explanation for the uncoupling of catabolism and anabolism under warming. Its direct temperature response is uncertain (depicted by the question mark) but we know it does respond strongly to substrate limitation.

One explanation for why maintenance- and growth-energy demands should remain coupled under warming for soil communities relies on the fact that differences in maintenance costs across species tend to co-vary with life histories. For example, resting metabolic rate, which we might think of as largely reflecting maintenance costs, increases with the temperature at which organisms live in cross-species syntheses (Clarke and Fraser, 2004; Clarke, 2006). Yet life histories also shift toward more active and more rapidly growing organisms as ambient temperature increases, and hence food intake rates are greater. So, maintenance costs increase because more active strategies are associated with higher intracellular enzyme and membrane (at least for eukaryotes) densities (Adadi et al., 2012). Yet these increases in maintenance costs plausibly increase proportionally with growth energy demands which are met by higher food intake, meaning that MGEs are invariant (**Figure 6**). For individual organisms, faster growth rates are often even associated with an increase in efficiencies because maintenance costs may be a relatively constant demand whether you are growing or not (Pirt, 1965; Ng, 1969). An increase in substrate availability and/or temperature then should increase MGEs, explaining the rise in efficiency of the unimodal temperature response observed for individuals (Crowther and Bradford, 2013). Notably, cross-species syntheses of resting metabolic rate (a proxy for maintenance energy) seem restricted to fish, which show the rise with environmental temperature, or with terrestrial insects, which conversely show a weak negative response of resting metabolic rate with temperature (Clarke and Fraser, 2004; Clarke, 2006). The evidential basis is weak, then, for varying maintenance costs to explain the decline in MGE with warming.

#### Energy spilling (waste metabolism)

So what physiological response can explain varying MGEs with temperature? The most likely explanation is “energy spilling” (reviewed in detail by Russell and Cook, 1995; Russell, 2007). This phenomenon encompasses a range of physiological pathways across different organisms, from bacteria to humans, and is also referred to as waste metabolism, spilling, uncoupling, overflow metabolism and waste respiration (Echtay, 2007; Russell, 2007). Use of the words “waste” and “overflow,” however, may be misleading because energy spilling may be beneficial. The benefits posited include: (a) resource interception, where even if a microbe can not grow it can prevent a competitor from doing so; (b) maintenance of a growth-ready (or metabolically alert) strategy, where energy acquisition can proceed in the absence of growth, and so be immediately available when conditions are favorable; (c) protection from toxins or charge differentials that arise from excessive metabolic activity, where energy spilling is essentially a safety valve; and (d) heat generation, energy spilling by bacteria can raise the temperature of biofilms above ambient and so presumably improve growth conditions (Russell, 2007; Tabata et al., 2013). Perhaps the best indicator that energy spilling is beneficial is the rapid death in certain environments of those microbes that do not energy spill (Russell, 2007).

In bacteria, energy spilling seems particularly high when the energy source (i.e., organic carbon) is in excess and nitrogen is strongly limiting (Russell and Cook, 1995). That is, where there is plenty of energy but no growth because of nutrient limitation. Conversely under carbon limitation, MGEs are ~10-times higher because catabolism and anabolism are again coupled (Schimel and Weintraub, 2003; Hackmann et al., 2013). These observations in pure culture make Frey et al.’s (2013) results even more intriguing: why were MGEs not higher in long-term warmed soils given the increased nitrogen availability (Rustad et al., 2001; Melillo et al., 2011)? We can also ask why MGEs on glucose were insensitive to temperature, when higher growth rates should have tipped energy demands proportionally toward anabolism? These questions highlight the difficulties of inferring how processes in culture translate to a complex environment such as soil, where a broad suite of growth strategies is represented. The majority, if not all, microbes in the soil use glucose. This ubiquitous use then likely aggregates a broad array of growth strategies, whereas more recalcitrant compounds are used by a small proportion of more specialized species (Hanson et al., 2008; Goldfarb et al., 2011). If traits such as storage of glycogen, which are associated with invariance in MGEs across environment, are differentially distributed across microbial groups then this could explain different MGEs for different substrates (Ng, 1969; Russell, 2007). We expect suites of traits to be related and so if glycogen storage is negatively related to extracellular enzyme production (Russell, 2007), then this and not the cost of extracellular enzyme production, which is relatively low (Allison et al., 2011), could explain declining MGEs with temperature on more recalcitrant substrates. Other explanations, such as differences in growth efficiencies between bacteria and fungi, or *r*- vs. *K*-strategies, now seem largely dismissed (Thiet et al., 2006; Strickland and Rousk, 2010).

Given uncertainties about the physiological mechanisms that determine MGEs, it is unlikely that we will be able to explain in the near term how they might adapt to warming. If energy spilling is beneficial, then under some environments (e.g., nitrogen limitation) reductions in MGE might even be adaptive! What seems likely is that substrate quality and availability, nutrient supply and microbial traits all contribute to observed MGEs. Direct warming effects on MGE are uncertain because conventional views of maintenance vs. growth energy demands fall short of explaining changing efficiencies with temperature. This means we may need to be satisfied with black-boxing the efficiency response of the soil microbial community to warming for current SOM modeling studies. The uncertainty also demands that we redress the paucity of observations we have for how warming affects MGEs of soil communities on specific substrates (Steinweg et al., 2008; Frey et al., 2013), where caveats such as changing substrate quality are controlled for. Culture-based studies can target specific mechanisms and should use isolates that are representative of soil decomposers because variation in microbial traits markedly influences how efficiencies respond to environment (see Russell, 2007).

#### Growth rates

Whereas MGE responses to temperature are far from clear, thermal adaptation in the growth of the soil microbial community resembles patterns expected from evolutionary trade-offs in the structure and function of both enzymes and membranes. That is, growth rates show the same unimodal temperature response as *R*_mass_ (**Figure 5**). These unimodal responses are shifted to the right under experimental warming (Bárcenas-Moreno et al., 2009; Rousk et al., 2012) and across spatial gradients in ambient temperature (Rinnan et al., 2009). These community-level responses match those for isolates of heterotrophic soil microbes (Crowther and Bradford, 2013), for aquatic microbial communities (Hall and Cotner, 2007) and for plants, vertebrates and invertebrates (Angilletta, 2009). These consistent, unimodal patterns suggest that trade-offs at the cellular-level translate to population and community performance (Angilletta, 2009).

Right-shifts in the unimodal growth response of soil microbial biomass to experimental warming are thought caused by species sorting (Bárcenas-Moreno et al., 2009), following the same explanation as for respiration (Bradford et al., 2010; Zhou et al., 2012). This sorting mechanism then explains the time taken (weeks to months) for these effects to manifest. The exception seems to be for very high temperatures; for example, Ranneklev and Bååth (2001) demonstrated that mimicking self-heating of peat by incubation at 55°C caused dramatic right-shifts in thermal optima for growth, resulting from the rapid growth of thermophilic bacteria. However, mesophilic and psychrophilic microorganisms take longer to grow (Ranneklev and Bååth, 2001), perhaps because they have to compete for resources with the more thermophilic organisms that can tolerate, at least for some time, cooler conditions. In contrast, at high temperatures, more thermophilc organisms may be able to grow unrestricted by competition because the original community is poorly adapted to the new temperature conditions. Whatever the mechanism, it seems likely that low growth rates do not permit species turnover within the time course of many cooling experiments (but see Curiel Yuste et al., 2010), explaining why shifts in optimum growth temperatures for communities are not observed under short-term cooling. What remains a mystery is why warming-induced phenotypic shifts in the individual physiologies of active soil microbes do not often translate to community-level processes at the same time-scale. For example, thermal adaptation in the growth and respiration of individual, mesophilic heterotrophic soil microbes occurs in just a few days (Crowther and Bradford, 2013) but shifts in community optima take weeks. Perhaps such responses are obscured from detection because of the host of other processes, such as desorption, that co-occur with warming (Subke and Bahn, 2010; Nie et al., 2013).

The consequences for SOM stocks of thermal adaptation in the growth rates of soil microbial communities have received little attention but may be minimal. Rousk et al. (2012) demonstrated that increases in the potential growth rates of soil bacteria were overwhelmed by reductions in growth rates caused by substrate depletion under experimental warming, meaning growth rates in control and warmed soils were essentially equivalent. Although not yet evaluated, I would argue that thermal adaptation of microbial *R*_mass_, growth rates and extracellular enzyme activity should accelerate the rate at which substrate depletion is achieved in warmed soils. That is, the theoretical “right shift” in these physiological parameters should lead to a microbial biomass that grows and degrades SOM more rapidly than non-adapted communities. How warming then influences substrate availability – through plant inputs, sorption/desorption and perceived chemical recalcitrance – therefore seems a key regulatory gate of SOM dynamics.

A key issue that I have not yet touched on, with regards measuring respiration and growth responses to temperature, is that we still have no direct methods for measuring soil microbial biomass and turnover (Bradford et al., 2009). We have many methods, including chloroform-fumigation extraction, substrate-induced respiration, total PLFA and semi-quantitative PCR, but all provide only correlated estimates of standing biomass (Wardle and Ghani, 1995). Estimates of turnover are even more uncertain. We should think about the uncertainty this generates in our observational and experimental data, and probably carry this forward into SOM and ecosystem models, to provide reliable error estimates for projected respiration and SOM stock responses to warming.

#### Biotic interactions

Heterotrophic soil microbes are part of a community that includes other microbes, such as arbuscular mycorrhizae and chemoautotrophs, as well as viruses, animals (e.g., Protozoa, nematodes, Collembola) and plants. These groups of organisms are faced with the same suite of physiological trade-offs in response to warming that heterotrophic microbes are (Van Dooremalen et al., 2013). Physiological responses of plants, animals and other microbes might influence soil microbial decomposer responses to warming but they are outside the purview of this review. Yet it is worth emphasizing that (a) temperature modulates the strengths of biotic interactions, and (b) interactions strongly determine the respiration, growth and community composition of soil microbes. For example, short-term increases in the overall growth of the soil microbial biomass under warming might be mitigated by concomitant increases in the growth of their predators, which in turn can promote microbial turnover and limit biomass. This microbial loop (sensu Clarholm, 1994) could explain increased availability of ammonium under experimental soil warming, but alternatively higher animal feeding can limit the growth and hence decomposer activity of heterotrophic microbes, as well as induce microbivore-defense, which represents a different energy cost (A’Bear et al., 2012; Crowther et al., 2012). Higher nitrogen availabilities could decrease fine root growth and exudation, limiting substrate available to soil microbes and shifting the soil community toward a more *K*-selected community (but see Zhou et al., 2012). Virtually no warming studies put microbial biomass responses in the full context of these biotic interactions, and yet we expect them to be major drivers of microbial activity.

## A PLACE FOR THERMAL ADAPTATION IN COUPLED CLIMATE-CARBON CYCLE MODELS

Implicit assumptions in conventional SOM models are that biological processes, such as respiration, conform to the principles of invariance, probability and simplicity (Bradford and Fierer, 2012). Such principles derive from classical physics and assume that past conditions do not influence future responses (invariance), that all organisms respond identically (probability), and that only a few, measurable variables influence outcomes (simplicity). Even if microorganisms are included as an SOM pool in the conventional models, they exert no control on respiration rates (Allison and Martiny, 2008). That is, if you removed the microbial pool, respiration would continue unabated because microbial activity is implicitly represented and donor-controlled. Specifically, respiration under this paradigm is represented as a first order reaction, where CO_2_ evolution from an SOM pool is a function of the pool size, and a decay rate constant that responds positively to temperature and moisture (Todd-Brown et al., 2012). Biological systems do not follow this paradigm because, in contrast to the principles of classical physics, organisms adapt to, differ in their tolerances of, and interact dependent on, environmental temperature. Such adaptive responses of organisms can, for example, scale to the level of ecosystem carbon exchange (Niu et al., 2012). Yet even when global convergence in the temperature sensitivity of ecosystem respiration was observed, Mahecha et al. (2010) cautioned that prescriptions of a constant Q_10_ value across systems was not justified. Instead they suggested that projections from coupled climate-carbon cycle models would be improved with a deeper understanding of the factors and processes affecting SOM mineralization.

Incorporating thermal adaptation and microbes into coupled climate-carbon cycle models is not, however, a straightforward exercise and the many challenges are reviewed elsewhere (e.g., Allison and Martiny, 2008; Ostle et al., 2009; Todd-Brown et al., 2012; Smith and Dukes, 2013; Todd-Brown et al., 2013). I wish to emphasize here only what I consider to be the major theoretical question related to incorporation of soil microbial processes and their responses to temperature. The question is: how best to represent soil microbes in models? I simplify this discussion by referring to SOM models and describe in the paragraph below the reason for focusing on these models, and in the subsequent paragraph elaborate on the question itself.

The land components of ESMs represent SOM dynamics relatively simply, but more complex representations are emerging. For example DAYCENT is the daily time-step version of CENTURY, one of the most widely used conventional SOM models, and is incorporated in version 4.5 of the land ecosystem model of the Community ESM (see Bonan et al., 2013). By considering SOM models we can then evaluate SOM responses that scale to local and global warming effects. At global scales SOM stocks are important in terms of a carbon store, whose loss might provide a positive feedback to climate change (Denman et al., 2007). At local scales, SOM stocks are inherently tied to ecosystem health because, for example, of the role SOM plays in preventing soil erosion, retaining moisture and nutrients, and providing soil structure and habitat (Lal, 2004).

The major theoretical question about representing soil microbes in models breaks down into two, broad choices: (a) as a supply driven pool, as in the conventional models; or (b) as a demand-driven pool, as in the new family of microbial SOM models? The primary difference between the two approaches is that the demand-driven approach creates a feedback between SOM turnover and microbial response (Allison and Martiny, 2008). In the conventional, supply driven approach microbes can be eliminated from the model and SOM continues to turnover. In the microbial SOM model approach, loss of the microbes brings SOM turnover to a halt because turnover is explicitly dependent on microbial activity. Representing the same microbial response to warming in the two different model structures can then have divergent consequences for SOM stocks. For example, a decline in MGEs with warming reduced SOM decomposition in the microbial SOM model of Allison et al. (2010), leading to no net change in SOM stocks. The same decline in efficiency, in contrast, led to greater losses of SOM for a conventional model (Frey et al., 2013). This decline occurred because in conventional models SOM decomposition rates are determined by temperature and formation rates by the assumed MGE. Hence warming translated to accelerated SOM decomposition, along with reduced formation rates because of declining growth efficiencies. In both model structures SOM formation rates are then a function of microbial growth, but the structures diverge because microbial activity explicitly regulates decomposition rates in the microbial SOM models but implicitly regulates it through temperature in the conventional models.

The conventional and microbial SOM model structures both assume that SOM turnover rates are dependent on microbial activity (Parton et al., 1988; Schimel, 2001; Lawrence et al., 2009; Allison et al., 2010). A third family of SOM models is required for hypothesis testing where only physico-chemical processes regulate SOM decomposition and formation rates (Kemmitt et al., 2008). My expectation is that such a family of models will be equivalent to neutral models: largely unrepresentative of what actually occurs (Clark, 2009; Warren et al., 2011b) but excellent at advancing our understanding of those processes that do regulate SOM turnover. I expect us to find that both biological and physico-chemical processes play important roles in SOM dynamics under warming, as argued by Conant et al. (2011) and as represented in conventional SOM models such as DAYCENT and RothC (Bonan et al., 2013).

## CONCLUSIONS

Thermal adaptation of organism respiration and growth rates should occur through fundamental evolutionary trade-offs in cellular physiology, such as between the structure and function of both enzymes and membranes. Individuals can adjust their physiology in response to sustained warming by producing warm-adapted isoenzymes and membrane structures, but changes in the physiology of the soil microbial biomass as a whole likely arise through shifts from colder- to warmer-adapted species (or at least genotypes). These physiological responses to warming are consistent with the idea that indirect warming effects, such as substrate depletion, at least partly explain apparent thermal acclimation of soil and ecosystem respiration to prolonged warming. Indeed, I hope that I have demonstrated in this review that thermal adaptation must occur in soil decomposer communities. As such, questions related to the consequences of thermal adaptation for carbon cycling must move from asking whether adaptation occurs, to asking what role adaptation plays in shaping ecosystem carbon stocks and flows in a warming world.

The idea that growth efficiencies of the soil microbial biomass decline with increasing temperature should be viewed as controversial. There is little empirical evidence that temperature directly elicits this response in soil communities and the physiological basis for the decline is not resolved. We should explore whether maintenance demands vs. energy spilling is the primary mechanism that uncouples anabolism and catabolism. Energy spilling seems more plausible but how it will adapt to warming is unclear because rather than “waste metabolism,” it likely has many fitness benefits for microbes. Uncoupling in favor of catabolism vs. anabolism causes declines in MGEs, which then prevent warming-induced SOM losses in microbial models and exacerbate them in conventional SOM models. Despite these divergent responses, both model structures assume that microbes regulate SOM turnover, an idea that has recently been questioned. Microbial ecologists thus face two challenges to the explicit incorporation of microbes in ESMs. We need to show conclusively that microbial activity does regulate SOM dynamics, and that adjustments in microbial physiology under warming can be represented in a manner commensurate with observed responses of soil respiration, microbial biomass and SOM stocks.

## Conflict of Interest Statement

The author declares that the research was conducted in the absence of any commercial or financial relationships that could be construed as a potential conflict of interest.
